# Gene Expression Browser: large-scale and cross-experiment microarray data integration, management, search & visualization

**DOI:** 10.1186/1471-2105-11-433

**Published:** 2010-08-20

**Authors:** Ming Zhang, Yudong Zhang, Li Liu, Lijuan Yu, Shirley Tsang, Jing Tan, Wenhua Yao, Manjit S Kang, Yongqiang An, Xingming Fan

**Affiliations:** 1 GeneExp, 310 South Third Street, San Jose, CA 95112, USA; 2 Institute of Food Crops, Yunnan Academy of Agricultural Sciences, Kunming 650205, Yunnan Province, China; 3 Biomatrix, Rockville, MD 20850, USA; 4 Vice Chancellor, Punjab Agricultural Univ., Ludhiana 141 004, India; 5 Plant Genetics Research Unit, ARS-USDA, at Donald Danforth Plant Science Center, 975 N. Warson Rd, St. Louis, MO 63132, USA

## Abstract

**Background:**

In the last decade, a large amount of microarray gene expression data has been accumulated in public repositories. Integrating and analyzing high-throughput gene expression data have become key activities for exploring gene functions, gene networks and biological pathways. Effectively utilizing these invaluable microarray data remains challenging due to a lack of powerful tools to integrate large-scale gene-expression information across diverse experiments and to search and visualize a large number of gene-expression data points.

**Results:**

Gene Expression Browser is a microarray data integration, management and processing system with web-based search and visualization functions. An innovative method has been developed to define a treatment over a control for every microarray experiment to standardize and make microarray data from different experiments homogeneous. In the browser, data are pre-processed offline and the resulting data points are visualized online with a 2-layer dynamic web display. Users can view all treatments over control that affect the expression of a selected gene via Gene View, and view all genes that change in a selected treatment over control via treatment over control View. Users can also check the changes of expression profiles of a set of either the treatments over control or genes via Slide View. In addition, the relationships between genes and treatments over control are computed according to gene expression ratio and are shown as co-responsive genes and co-regulation treatments over control.

**Conclusion:**

**Gene Expression Browser **is composed of a set of software tools, including a data extraction tool, a microarray data-management system, a data-annotation tool, a microarray data-processing pipeline, and a data search & visualization tool. The browser is deployed as a free public web service (http://www.ExpressionBrowser.com) that integrates 301 *ATH1* gene microarray experiments from public data repositories (viz. the Gene Expression Omnibus repository at the National Center for Biotechnology Information and Nottingham Arabidopsis Stock Center). The set of Gene Expression Browser software tools can be easily applied to the large-scale expression data generated by other platforms and in other species.

## Background

A microarray measures the expression of thousands of genes simultaneously. This experimental system has revolutionized biological research by enabling discovery of a large set of genes whose expression levels reflect a given cell type, treatment, disease or development stage. Since the advent of this technology more than a decade ago, a large amount of expression data has been accumulated on more than 100 species [1]. Several initiatives have been undertaken to develop microarray public data repositories and analysis tools for scientists to share and utilize these data [2]. The public data repositories, such as NASC, NCBI GEO [3], EBI ArrayExpress [4,5] and NIG CIBEX [6], have been collecting, annotating, storing and redistributing large amounts of microarray data from diverse experiments. For example, NCBI GEO (http://www.ncbi.nlm.nih.gov/geo/) has collected 366,965 samples from 14,304 experiments. These microarray data are invaluable resources for scientific research and discovery.

Effective utilization of these datasets has, however, been limited because of a shortage of suitable tools to integrate large-scale and diverse microarray datasets. In most common use case, a scientist performs an experiment-based analysis: he or she downloads microarray data and sample annotations corresponding to a single experiment, inputs the data into a microarray data-analysis tool, such as GeneSpring [2], HDBStat! [7], or Bioconductor packages [2], etc., and carries out single-experiment centered analysis. In another common use case (e.g. for many gene-centric studies), a scientist wants to know how the expression of a given gene changes under various experimental conditions. The latter case is critically important for discovering gene functions, validating biomarkers, and developing new drugs targeted to specific genes. To answer gene-centric questions, we must have a tool that can be used to integrate a large amount of data from different microarray experiments. Developing such a tool presents several challenges.

The first challenge is the heterogeneity of data collected from different microarray experiments. Different microarray experiments from different laboratories are usually designed independently for specific research purposes. Heterogeneity might come from differences in experimental designs, materials sampled, developmental stages, treatment levels (including controls), and so on. The second challenge is to develop an effective software tool to process such a large amount of data at an acceptable speed with currently available hardware resources (i.e., CPU, memory and network). The third challenge is related to the complexity of displaying or visualizing data in a software tool. Most software tools, when applied to large data sets, display items in an extended page or multiple display pages. Therefore, it is impossible for users to get an overall view of the data on a single page. It is also inefficient and inconvenient for users to scroll display pages to find interesting information from thousands of data items. Thus, it is important to design a data display interface that can show both an overall view of a large-scale dataset in its totality and a detailed view of individual data points.

Genevestigator [8] and GeneChaser [1] are two web-based gene expression visualization tools that have successfully integrated a large number of microarray datasets and facilitated gene-centric and cross-experiment gene-expression discoveries. Genevestigator defines experiment annotation categories as Tissues/Organs, Developmental Stage, Environmental Factors (Stimulus) and Mutation. The expression data and the analysis results are organized according to these categories. The microarray experiments are discarded if they cannot be classified into one of the predefined categories. GeneChaser, on the other hand, automatically re-annotates and analyzes GDS datasets from NCBI GEO. It segregates all experimental conditions (treatment levels) into groups and then performs group versus group comparisons. However, the display systems of both Genevestigator and GeneChaser are limited. These two tools display data with heatmap or bar graphics on a display page with extended dimension or in multiple display pages. Only a limited number of data points can be shown at a time. Users have to scroll down the page to find interesting data points from among hundreds or thousands of total experimental conditions.

The GEB, on the other hand, displays efficiently a large number of data points simultaneously. This has been achieved by developing a set of software tools of data extraction, data management, data annotation, data processing, and gene expression profile search & visualization. This set of software tools can be applied to microarray data in both public and private data repositories. The current public GEB web service (http://www.ExpressionBrowser.com) integrates 301 ATH1 microarray experiments that were originally stored in the data repositories of NCBI and NASC [9]. *Arabidopsis*, as a model plant, is widely used in various microarray experiments and gene-network modeling [10-12]. The results and knowledge obtained from *Arabidopsis *studies can be used as a reference for corresponding research on other plants, especially field crops [13,14].

## Implementation

### Overall design of workflow

**Figure 1:**
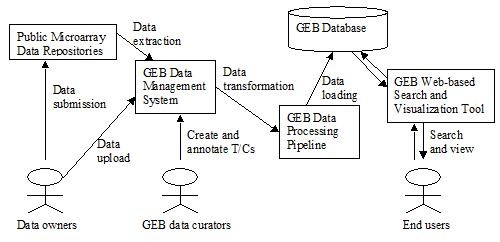
**The schema and workflow of GEB**. Microarray data can be downloaded into GEB from public repositories or uploaded into GEB by data owners. GEB is composed of a set of functional components. The major components are the data extractor (a command-line program), the data management system (a web application), the data processing pipeline (a set of command-line programs piped together), a MySQL database, and a web-based search and visualization tool.

The GEB workflow is shown in Figure 1. Microarray data can be downloaded from public data repositories with the data extraction tool. Alternatively, data owners may upload their data directly into GEB. The data extraction tool harvests raw data files, sample annotations, and experimental designs from data repositories into the GEB data-management system. Data curators use the web-based interfaces of the data-management system to create sample sets by combing all replicated samples in each treatment level into individual groups (i.e. sample sets). Then, the data curators define a T/C by selecting a treatment sample set and a control sample set. In the data-processing pipeline, the microarray data are normalized, and the log2 ratio of treatment-over-control (LOG2R) and its t-test *P *value are calculated. The normalized intensities of each chip, average intensities of each sample set, LOG2Rs and *P *values of each T/C are loaded into the GEB database, from which the data can be queried via the web-based search & visualization tool.

### Affymetrix probe set annotation

The probe sets on Affymetrix *ATH1 *chip were annotated via the following procedures: (1) *Arabidopsis *cDNA sequences and annotations were downloaded from TAIR (http://www.arabidopsis.org/) and *ATH1 *probe sequences were downloaded from Affymetrix; (2) All probe sequences were BLASTed against all cDNA sequences; (3) A probe set was mapped to a cDNA when nine or more probes in the probe set had a 100% match to a cDNA sequence (each *ATH1 *probe set contains 11 probes); and (4) The annotation of matched cDNA was used as the annotation of the probe set.

### Data extraction and management

The data extraction tool was developed using Java with Jakarta Commons Net Library (http://commons.apache.org/net/). The tool is a web crawler that recursively harvests raw data (such as Affymetrix CEL files), sample annotations, and experiment design descriptions from a repository website and then loads them into GEB database. To download data from different repositories, a corresponding plug-in component was developed for each repository. So far, two data extraction plug-ins have been developed for harvesting data from GEO and NASC.

**Figure 2:**
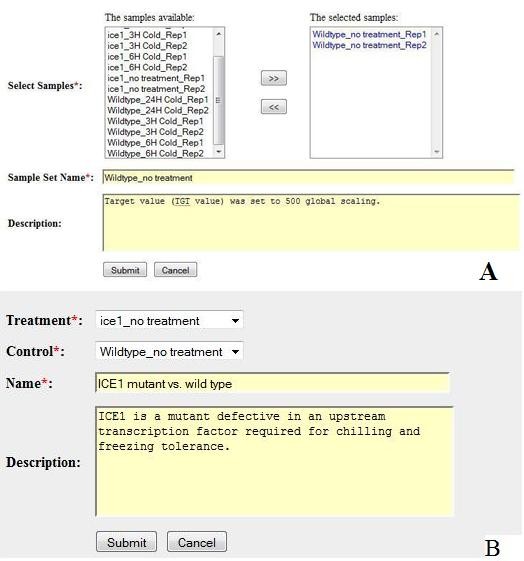
**Screenshots of the GEB data management system**. A. The web interface used by a data curator to define sample sets for all experiment data. B. The web interface used by a data curator to choose a treatment sample set and a control sample set to create a T/C. Detailed information for all eight sample sets can be found at http://expressionbrowser.com/arab/displayExperiment.jsp?id=2202517&tab=2.

The data-management system was developed for data curators to view and annotate the microarray data extracted from data repositories or submitted by data owners. Data curators annotate the data via the following steps:

First, a data curator creates a sample set by grouping replicated samples from every treatment level. The user interface for defining a sample set is shown in Figure 2A. A sample set name of "Wildtype_no treatment" is given at Name box and two replicates of "Wildtype_no treatment_Rep1" and "Widetype_no treatment_Rep2" are assigned to the sample set by moving them from the left panel to the right panel. Other sample sets in the experiment are created via the same procedure as noted above.

Second, a data curator creates a T/C pair by choosing a treatment sample set and the corresponding control sample set from a drop-down menu (Figure 2B). For instance, we selected "ice1_no treatment" as treatment and "Wildtype_no treatment" as control to form a T/C. Then, the curator specifies a name of "ICE1 mutant vs. wild type" at Name box and detailed T/C information is given in Description box at the lower panel of Figure 2B. The control sample set is selected for a given treatment sample set so that only one-factor differs between the treatment and the control. Therefore, the biological effect of the T/C will be clearly distinguished by the differential factor. All possible T/C pairs were created in this way. In the example shown in Figure 2, a total of 10 T/Cs are defined as follows: 3 T/Cs for cold effects in a mutant (viz. "Ice1 mutant with cold treatment for 3 hr vs. Ice1 mutant with no treatment", "Ice1 mutant with cold treatment for 6 hr vs. Ice1 mutant with no treatment", and "Ice1 mutant with cold treatment for 24 hr vs. Ice1 mutant with no treatment"); 3 T/Cs for cold effects in wild type (viz. "Wildtype with cold treatment for 3 hr vs. Wildtype with no treatment", "Wildtype with cold treatment for 6 hr vs. Wildtype with no treatment", and "Wildtype with cold treatment for 24 hr vs. Wildtype with no treatment"); 3 T/Cs for mutation effects under cold treatment (viz. "Ice1 mutant with cold treatment for 3 hr vs. Wildtype with cold treatment for 3 hr", "Ice1 mutant with cold treatment for 6 hr vs. Wildtype with cold treatment for 6 hr", and "Ice1 mutant with cold treatment for 24 hr vs. Wildtype with cold treatment for 24 hr"); and one T/C for mutation effects without cold treatment (viz. "Ice1 mutant with no treatment vs. Wildtype with no treatment"). All 10 T/Cs are shown at http://expressionbrowser.com/arab/displayExperiment.jsp?id=2202517&tab=1. After all treatment levels in each experiment are transformed into T/Cs, different experiments have same data structure and are comparable to one another and are, thus, easily integrated together. As a result, the heterogeneity caused by the differences in experimental designs is removed. The LOG2R of T/C also removes system errors that affect both treatment and control. Therefore, the ratio data generated based on T/Cs can be more instructive and reliable than intensity data generated from treatment levels.

### Data processing and data quality monitoring

**Figure 3:**
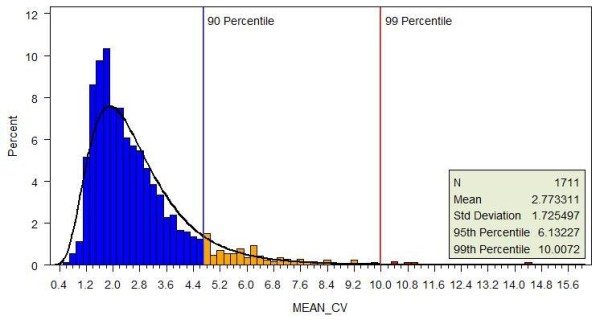
**The distribution of mean %CV of all sample sets**. The mean %CV is calculated in two steps: first calculate the standard deviation, mean, and %CV of each gene in a sample set (%CV = 100 * Mean intensity/Standard deviation), and then compute the mean %CV of all genes in the sample set.

The GEB data-processing pipeline is composed of four consecutive programs. The first program is for data normalization using the Robust Multichip Average (RMA) algorithm [15] that was implemented in the Bioconductor Affy package (http://www.bioconductor.org/packages/2.4/bioc/html/affy.html). The second program takes this normalized intensity data as input and computes average intensities, standard deviations, LOG2Rs, and *P *values of two-sample, two-tailed t-tests. The third program renders JPEG images of MA plots [16,17] with average intensity as the x-axis, LOG2R as the y-axis, and *P *value as the color. The images are loaded into the GEB application server (Tomcat) for data display when queried by users. The fourth program computes the mean percentage coefficient of variation (%CV) of all microarray features (genes) in a sample set using the following two steps. First, the standard deviation, mean, and %CV of each feature (gene) in a sample set are calculated: that is, %CV = 100 * (Mean intensity/Standard deviation). Second, the mean %CV of all features in the sample set is calculated. The mean %CV of each of individual sample set is computed via the above procedure; the distribution of all mean %CVs is shown in Figure 3. Most sample sets have mean %CV between 0.5 and 4.68. There is a long tail to the right side of the distribution, in which the mean %CV ranges from 4.68 to 16. This result indicates that about 10% of the total sample sets have extremely large mean %CV, and thus probably have poor data quality. Mean %CV of a sample set could be used to monitor quality of the sample set because higher mean %CV implies larger variation among the replicated samples in the sample set. Therefore, any finding or conclusion from a sample set with high mean %CV must be interpreted cautiously. We plan to filter out the sample sets with extremely high mean %CV in the future to guarantee the quality of all the data in GEB.

Some microarray experiments in NASC or GEO were discarded because there were no replicated samples or no suitable controls. As of now, there are a total of 301 experiments, 1450 T/Cs, and 33,074,500 LOG2R data points in the *Arabidopsis *GEB database. Additional data, when available, can be easily entered into GEB.

### Data search and visualization

The Lucene search engine (http://lucene.apache.org/) is used for full-text search. Search index files in GEB are built with the text from gene identifiers, gene symbols, gene annotations, T/C names, T/C descriptions, experiment titles, and experiment descriptions. Genes, T/Cs, and experiments are searchable by matching keywords in the index files.

A 2-layer visualization display is designed to show large-scale data points as both an overall view and a detailed view. This visualization was developed using AJAX technology [18]. The first display layer is a static display (image) generated offline that contains all data points. The second layer is a real-time interactive display built by Web2.0 technology (JavaScript/AJAX). With the 2-layer display, users not only obtain an overall expression profile of the distribution of data points on the static plot, but can also get detailed information on each data point by real-time interactive searching or highlighting. The *P *value of ratio data is shown by the color of the data. Therefore, data significance level is displayed at the same time as the magnitude of the data is.

## Results and Discussion

### Full-text search

With full-text searching, users can easily access the information inside GEB. The full text searching method employed by GEB is different from the searching in Genevestigator [8] or GeneChaser [1], in which only gene identifiers or symbols can be used for searching. Users can obtain expression information from Genevestigator or GeneChaser only when they clearly know the gene names or symbols. In contrast, GEB carries out full-text search for any word or letters for a gene symbol, gene annotation, T/C name, T/C description, experiment title and experiment description. Users can freely explore the expression data with any search term they wish.

**Figure 4:**
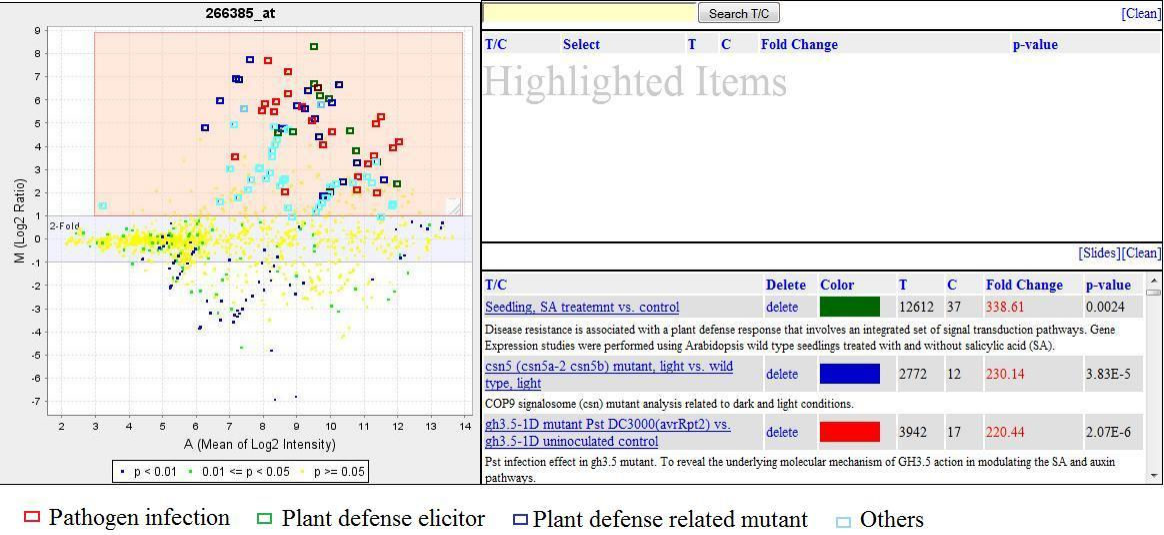
**The Gene View of *PR-1 *that is a disease-related gene**. The up-regulation T/Cs were highlighted and selected. The color can be changed by right clicking on the color icon in the lower box of right panel in the figure. Users may test this functionality at http://expressionbrowser.com/arab/displayFeature.jsp?id=1001343.

**Figure 5:**
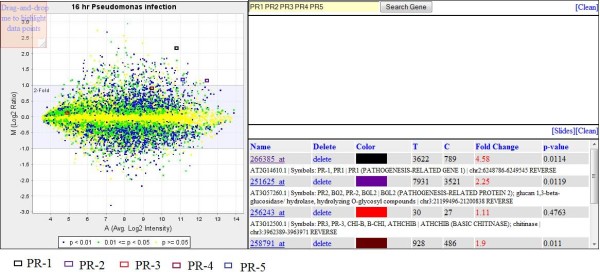
**The T/C View of "16 hr Pseudomonas infection"**. When *PR1, PR2, PR3, PR4 and PR5 *were searched and selected on this T/C View, the data points on the MA plot on left panel are labeled with a colored box. The color can be changed by right clicking on the color icon on the lower box of the right panel in this figure. You may test this function at http://expressionbrowser.com/arab/displayPair.jsp?id=2056966.

The full-text search is implemented in three places. The first is the GEB home page (http://www.ExpressionBrowser.com), where the user can enter keywords and find three types of information: genes, T/Cs and experiments. The second place is in Gene View (Figure 4), where users can search T/Cs and investigate how different T/Cs affect the expression of the selected gene. The third place is in the T/C View (Figure 5), where users can search genes and observe how the expressions of these genes are changed by the selected T/C.

### Gene View and co-responsive genes

The GEB backend data model is a matrix with two dimensions, genes and T/Cs. Users visualize the expression profiles as a slice along either of these two dimensions: the Gene View displays data points of all T/Cs for a selected gene, whereas the T/C View displays data points of all genes for a selected T/C.

Figure 4 illustrates the Gene View. Data points from all T/Cs for a gene are displayed in the MA plot [16,17]. Here, M, the y-axis, is the log2 ratio of treatment over control (LOG2R) [log2 (treatment intensity) - log2 (control intensity)] and A, the x-axis, is the average log2 intensity of the treatment and control [(log2 (treatment intensity) + log2 (control intensity))/2]. The MA plot provides a quick overview of data points for all T/Cs affecting the selected gene. The data points located in the upper area of the MA plot are 'up-regulation' T/Cs, and those located at lower area are 'down-regulation' T/Cs. Gene View is a cross-experimental display of the expressions of a gene under all experimental conditions currently available in GEB. With the MA plot, users can get a clear overall view of a gene-expression profile without scrolling down the display page, no matter how many data points might be on the plot.

From a GEB MA plot, users can easily view both the LOG2R changes and also the statistical significance of the LOG2R. Each data point is color coded on the basis of the t-test *P *value that indicates the significance level of its LOG2R. The data points are coded in blue color when *P *values are lower than 0.01, in green color when *P *values are between 0.01 and 0.05, and in yellow color when *P *values are higher than 0.05. The color-coded data points help users know visually significance levels and reliability of the data. For example, if the data point has both a high-fold change (at the top or bottom of the display) and high *P *values (*P *> 0.05, yellow color), it suggests that there may be large systematic or experimental errors among replications so that the results should be interpreted cautiously before conclusion are drawn based on such a data point. Therefore, the location and color of the data points on the GEB MA plot give users a clear view of gene expression in both ratio scale and significance level (reliability).

The MA plot is a JPEG image generated by the offline data-processing pipeline. The image is about 60 K in size, with 480 × 480 pixel dimensions, which allows the image to be loaded from host server to users' browser very quickly so that users can rapidly obtain an overall view of the expression profile of a gene. Most importantly, GEB is equipped with highlighting and search functions that allow users to highlight data points by dragging-and-dropping the mouse and to search data by entering keywords. Figure 4 illustrates how to use the "highlighting window" to locate the up-regulation T/Cs on the MA plot. First, users move the "highlighting window" to cover the data points on the upper panel of the MA plot. The users can resize the window, if needed. The two text boxes to the right of the MA plot are used for listing detailed information about the highlighted data points. Users can click the 'Select' button for any T/C on the upper text box and then the selected T/C will be moved to lower text box. At the same time, the selected T/C is also marked on the MA plot with a small rectangle. This two-layer display solution achieves both a quick overview of an expression profile and a detailed view of the selected data points.

*Arabidopsis PR-1 *gene, a pathogenesis-related gene [19], was used as an example of Gene View in Figure 4. The up-regulation T/Cs selected in Figure 4 are listed in Table 1. A total of 95 T/Cs were selected when 2-fold and P < 0.05 were used as a double cutoff. Among the 95 T/Cs, 44 T/Cs are pathogen treatments, 13 T/Cs are plant defense elicitor treatments, and 14 T/Cs are plant defense-related mutants. These results clearly suggested that the expression of *PR-1 *was promoted by infections, plant-defense elicitors, and plant defense-related mutations. In previous studies, *PR-1 *was defined as a pathogenesis-related gene that was coordinately activated by pathogen infection and functioned as an indicator of the defense reaction [20,21]. The silencing of this gene leads to an increase in extracellular β-(1→3)-glucanase activity at the onset of tobacco defense reactions [22-24]. A decrease in β-(1→3)-glucan deposition in *PR-1*-silenced lines [22] might cause less deposition of callose that is linked with β-(1→3)-glucan and while the callose deposition is one of the characteristics of defense reactions associated with hypersensitive response of a plant [25]. Morris et al. [26] indicated that chemical induction of maize *PR-1 *genes increased resistance to downy mildew. The results for *PR-1 *functions revealed by GEB were impressively consistent with the previous findings. These results strongly suggested that Gene View of GEB would be very useful in gene-function discovery, biomarker validation, and bioprocess identification.

**Table 1:** A list of T/Cs that induces the expression of the *Arabidopsis PR-1 *gene.

T/C Name	Treatment type	Fold Change	P-value
Seedling, SA treatment vs. control	Plant defense elicitor	338.61	0.0024

Csn5 (*csn5a-2 csn5b*) mutant, light vs. wild type, light	Plant defense related mutant	230.14	3.83E-05

*gh3.5-1D *mutant Pst DC3000(avrRpt2) vs. *gh3.5-1D *un-inoculated control	Pathogen infection	220.44	2.07E-06

Leaf, *eds16 *mutant, *Golovinomyces orontii *infection for 7 d vs. *eds16 *mutant, 0 d control	Pathogen infection	160.28	2.21E-05

*Csn4-1 *mutant, light vs. wild type, light	Plant defense related mutant	130.35	6.81E-04

*Csn4-1 *mutant vs. wild type	Plant defense related mutant	125.67	0.0011

BTH Effect for 24 hr in *wrky18 *mutant	Plant defense elicitor	111.62	0.0012

Whole plant, *mkk1*/*mkk2 *vs. WT	Plant defense elicitor	108.59	5.48E-04

senescence effects in pod	Senescence	97.64	2.67E-05

*cpr5scv1 *double mutant	Plant defense related mutant	88.61	0.0385

Pst DC3000 infection (12 hr) in WT	Pathogen infection	83.37	0.0181

BTH Effect for 24 hr in WT	Plant defense elicitor	77.47	0.012

Whole plant, WT, 24 h BTH vs. WT control	Plant defense elicitor	71.71	3.44E-07

*Csn3-1 *mutant, light vs. wild type, light	Plant defense related mutant	67.19	7.58E-05

120 hr *Erysiphe orontii *infection	Pathogen infection	64.8	0.0053

Whole plant, *mkk2*, 24 h BTH vs. *mkk2 *control	Plant defense elicitor	63.45	0.0042

Col-0 WT, Pst DC3000 (avrRpt2) infection vs. un-inoculated control	Pathogen infection	60.32	0.0182

Cold 7 days effects	Others	58.93	0.0061

*cpr5 *mutant	Plant defense related mutant	56.63	0.0354

Pst DC3000 infection (12 hr) in *wrky17 *mutant	Pathogen infection	55.62	0.0293

*siz1-3 *mutant drought with treatment vs. Col-0 WT with drought treatment	Plant defense related mutant	52.98	0.0034

*Brm-101 *mutant vs. Ler WT	Others	52.5	0.0221

96 hr *Erysiphe orontii *infection	Pathogen infection	49.32	2.50E-05

*Phytophthora *infection for 24 hr	Pathogen infection	47.65	3.19E-05

32 hr PsES4326 infection vs 9 hr PsES4326 infection	Pathogen infection	41.11	0.0267

*siz1-3 *mutant vs. Col-0 WT	Plant defense related mutant	38.88	0.0031

Pst DC3000 infection (12 hr) in *wrky11 *mutant	Pathogen infection	37.07	0.0093

24 hr PsES4326 infection vs 9 hr PsES4326 infection	Pathogen infection	33.64	0.0297

E2Fa-DPa over-expressing	Others	32.75	0.009

Cotyledon	Others	30.2	8.69E-05

Chitin receptor mutant, chitooctaose treatment vs. Wild type, chitooctaose treatment	Plant defense elicitor	29.64	7.04E-04

shoot vs root	Others	29.61	8.02E-04

*Csn5 *(*csn5a-2 csn5b*) mutant, dark vs. wild type, dark	Plant defense related mutant	29.26	0.007

Chitin receptor mutant vs. Wild type	Plant defense related mutant	28.92	3.09E-05

flower stage 15, sepals	Others	28.05	1.08E-04

Whole plant, *mkk1*, 24 h BTH vs. *mkk1 *control	Plant defense elicitor	26.74	0.0174

Leaf, WT, *Golovinomyces orontii *infection for 7 d vs. 0 d control	Pathogen infection	26.06	2.36E-07

BTH Effect for 8 hr in WT	Plant defense elicitor	26.06	0.0201

BTH Effect for 8 hr in wrky18 mutant	Plant defense elicitor	25.92	3.30E-04

*camta3-2 *mutant vs. wild type	Plant defense related mutant	22.87	0.0358

*cdpk6-yfp 4 *transgene effects	Others	20.98	0.0151

PsmES4326 infection for 32 hr	Pathogen infection	19.53	0.0079

Leaf, WT, *Golovinomyces orontii *infection for 5 d vs. 0 d control	Pathogen infection	17.73	2.80E-06

*S15-118 *mutant vs. WT	Others	17.6	0.0368

PsmES4326 infection for 24 hr	Pathogen infection	16.37	0.0072

flower stage 15	Others	14.82	1.09E-04

BTH treatment in WT vs. WT control	Plant defense elicitor	14.78	3.70E-04

*Pseudomonas syringae *pv *phaseolicola *infiltration for 24 hr	Pathogen infection	12.87	0.0015

mature leaves, 35 days after sowing vs. Average	Others	12.58	0.0232

72 hr *Erysiphe orontii *infection	Pathogen infection	12.55	0.0086

old rosette leaf vs young rosettet leaf in WT	Senescence	10.82	0.0235

*SPH1 *knockout vs WT in young rosette leaf	Others	10.69	0.0187

*pmr5 pmr6 *double mutant vs. WT	Plant defense related mutant	10.46	0.0293

*Pseudomonas syringae *pv *tomato *avrRpm1 infiltration for 24 hr	Pathogen infection	10.19	0.0036

flower stage 12 equivalent (7)	Others	8.83	3.61E-04

*sni1 *mutant	Others	8.59	0.0117

flower stage 12 equivalent (6)	Others	8.58	2.48E-04

High nitrogen and glucose effects	Others	7.64	0.0015

*Pnp1-1*, phosphate deficiency 1 wk vs. WT, phostphate deficiency 1 wk	Others	6.92	0.0259

*Pseudomonas syringae *pv *tomato *DC3000 hrcC-infiltration for 24 hr	Pathogen infection	6.78	1.75E-04

glucose effects	Others	6.42	7.38E-04

*Pnp1-1*, phosphate efficiency 1 wk vs. WT, phostphate efficiency 1 wk	Others	6.36	0.0173

*mil4 *overexpression line with BTH treatment vs. *mil4 *overexpression line control	Plant defense elicitor	6.3	5.77E-04

flower stage 12, sepals	Others	6.2	3.95E-04

arr10 arr12 double null mutant effects under cytokinin	Others	6.03	0.0034

*pmr5 *mutant vs. WT	Plant defense related mutant	5.88	0.0412

WT, INA 48 h vs. control 48 h	Others	5.77	0.0437

*pnp1-1 *mutant, phosphate starvation for 1 wk vs. pnp1-1 mutant, 1 wk control	Others	5.74	0.0402

BTH treatment in mil4 mutant vs. H2O in mil4 mutant	Plant defense elicitor	5.52	0.0028

Cotyledon	Others	5.46	3.86E-04

WT, phosphate starvation for 1 wk vs. 1 wk control	Others	5.28	0.0166

seedling 3 vs average	Others	5.1	8.30E-04

seedling 2 vs average	Others	4.87	0.001

*SAM SE*, 35S:AGL15 vs. WT	Others	4.64	0.0461

16 hr Pseudomonas infection	Pathogen infection	4.58	0.0114

gl1T rosette leaf #4, 1 cm long	Others	4.53	1.56E-04

*Pseudomonas syringae *pv *phaseolicola *infiltration for 6 hr	Pathogen infection	4.33	0.0308

senescing leaves	Senescence	4.32	3.95E-05

*Botrytis cinerea *infection on 48 *hpi *leaf	Pathogen infection	4.17	0.0247

Col-0 rosette leaf #4	Others	4.08	0.0016

*mil4 *mutant vs. WT	Plant defense related mutant	3.81	0.0031

*gl1T *rosette leaf #12	Others	3.64	3.24E-04

flower stage 12 equivalent (5)	Others	3.62	0.0028

Leaf	Others	3.22	0.0016

cauline leaves	Others	3.13	0.0023

shoot under potassium starvation	Others	3	0.0098

*Met1-3 *mutant leaf (4th generation) vs. Col-0 WT	Others	2.9	0.0353

shoot under Caesium treatment	Others	2.9	0.0061

Col-0 rosette leaf #4	Others	2.76	0.0018

24 hr control vs 0 hr control	Others	2.7	0.0158

Ambient CO2 and Ambient Light at 96 hr vs 0 hr	Others	2.49	0.0364

rosette leaf # 2	Others	2.45	0.0075

leaf 7, distal half	Others	2.42	0.0023

*HSP90 *reduced mutant (RNAi-B1) vs. Control-3	Others	2.05	0.0063

*gh3.5-1D *mutant, Pst DC3000 (avrRpt2) infection vs. Col-0 WT, Pst DC3000 (avrRpt2) infection	Others	2.03	0.0263

**Figure 6:**
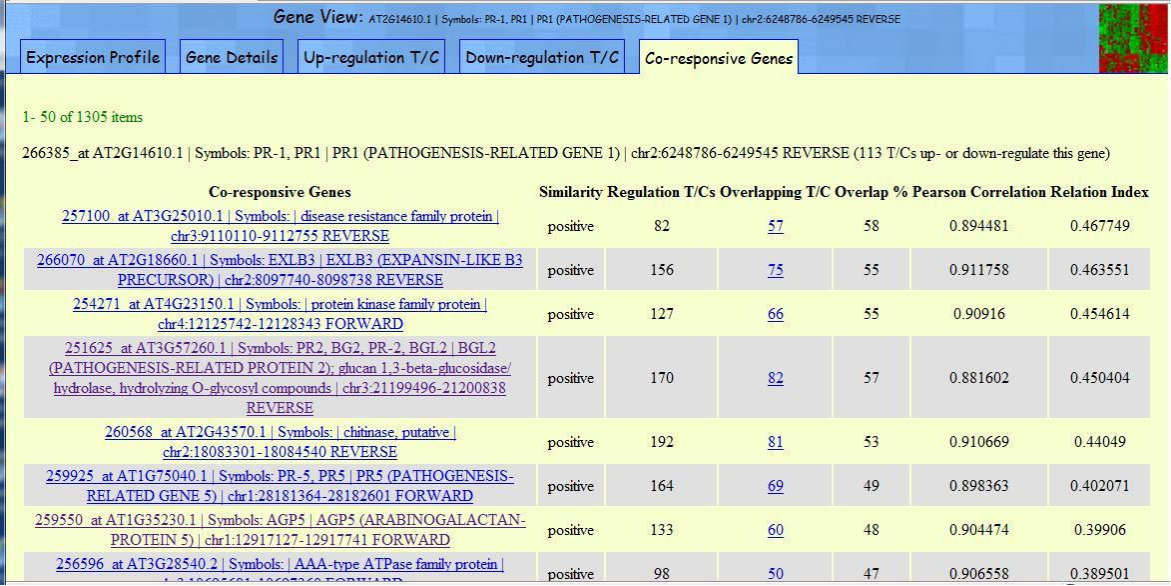
**The co-responsive genes related to *PR-1***. The co-responsive genes were listed in the order of their relation index to *PR-1 *genes. The relation index is a product of the overlap percentage (the percentage of overlapped co-regulation T/Cs between *PR-1 *and the selected gene) and the correlation coefficient (the Pearson correlation coefficient among the overlapped T/Cs). More co-regulation T/Cs can be found at http://expressionbrowser.com/arab/displayFeature.jsp?id=1001343&tab=4.

Figure 6 represents a screenshot of "Co-responsive Genes" tab in the *PR-1 *Gene View (http://www.expressionbrowser.com/arab/displayFeature.jsp?id=1001343&tab=4). The co-responsive relationship of two genes is determined by the following procedure: (1) The up- and down-regulation T/Cs of the two genes are selected using a double cutoff of *P *< 0.05 and of 2-fold; (2) the overlap T/Cs that have the two genes selected are then used to compute the overlap percentage; (3) the Pearson correlation coefficient is calculated using the LOG2R of overlapped T/Cs; and (4) a relationship index is calculated using the overlap percentage multiplied by the square of the correlation coefficient. The relationship between the two co-responsive genes is computed with ratio data from T/C with only a single factor differing between treatment and control. Therefore, the relationship between co-responsive genes solely reflects the effect of a biological treatment because the variations caused by most other factors are removed. On the other hand, if the relationship between co-expressed genes is computed with intensity data where multiple factors vary (such as tissue and cell type of sample, biological treatment, sampling methods, such as time and location, experimental methods, such as sample storage, mRNA extraction, or microarray dying, and systematic errors), then the relationship between co-expression genes reflects the mixed effects from biological treatment and these multiple factors. In the list of *PR-1 *co-responsive genes (Figure 6), impressively, many well-known plant defense-related genes, such as *EXLB3, PR-2, Chitinase, PR-5 *and *AGP5*, were found. Among them, *PR-2 *and *PR-5 *are considered to have a similar function as *PR-1 *in systemically acquired resistance (SAR) responses [27]. According to a review on the integrated application of online data mining tools by Meier and Gehring [28], *PR-1*, *PR-2 *and *PR-5 *were induced by necrotrophic *Botrytis cinerea *pathogen. The results shown by GEB are consistent with those from previous studies. The consensus results from multiple experiments in GEB provide reliable clues for gene-expression discoveries.

### T/C View and co-regulation T/Cs

Figure 5 represents an example of T/C View of "16 hr Pseudomonas infection." Each data point on the T/C View is the LOG2R of a gene. The MA plot, color codes, two-layer display design, and searching/highlighting functions on the T/C View are exactly the same as those in Gene View described above. The following example shows how to use search function to locate genes in the T/C View. When a string of "*PR1 PR2 PR3 PR4 PR5*" was used as a search keyword, all genes with any matching word in its annotation are shown in the upper right box (Figure 5). By clicking the 'Select' button on each gene, the gene is moved to the lower box. At the same time, the selected gene is marked on an MA plot with a small rectangle. T/C view provides a condition-centric view of microarray data.

Though different T/Cs may stimulate different sets of genes, any two different T/Cs may co-regulate a set of genes such that they have similar gene-expression signatures. The co-regulation relationship between two T/Cs can be constructed from the similarity of gene-expression signatures of the two T/Cs. If we click the "Co-regulation T/Cs" tab in the T/C View of "16 hr Pseudomonas infection" (http://expressionbrowser.com/arab/displayPair.jsp?id=2056966&tab=4), a total of 199 co-regulation T/Cs are listed in a table ordered by their "relation index" to the "16 hr Pseudomonas infection" T/C. The calculation of relation index between the two T/Cs is described in the footnote in Table 2. The T/C of "24 hr Pseudomonas infection" has the closest relationship (with relation index of 0.623816) to "16 hr Pseudomonas infection." This result is easily understood because they are the same treatment with an 8-hour treatment-time difference. The top 80 (of the 199) co-regulation T/Cs of "16 hr Pseudomonas infection" are listed in Table 2: 29 belong to pathogen-infection, 16 are plant-defense elicitors, and 6 are plant defense-related mutants. It is interesting to note that 3 T/Cs are negatively correlated with the T/C of "16 hr Pseudomonas infection" (Table 2). Two of the three T/Cs are mutants of Enhanced Disease Susceptibility 16 (*EDS16*) under infection conditions. *EDS *genes have special function in basal disease resistance to pathogens as well as *R *genes [29,30]. *Arabidopsis EDS *mutants, such as *eds1 *[31] and *eds5 *[32], have lower PR gene-expression level and exhibit higher susceptibility to pathogen infection. The reverse relationship of gene-expression signatures between *EDS16 *under infection and "16 hr Pseudomonas infection" implies that some pathogen-related genes are either not activated or reduced in *EDS16 *mutants when they are infected by pathogens. Another T/C negatively correlated with the "16 hr Pseudomonas infection" is caused by "high nitrogen effect". Hoffland et al. [33] reported that high nitrogen application caused higher N concentration in plant tissue, and the effect of tissue N concentration on disease susceptibility was highly pathogen-dependent. They found that disease susceptibility to *P. syringae *and *Oidium lycopersicum *was significantly increased with increasing N concentration in tomato tissue [34]. The results obtained from GEB are consistent with the previous independent studies, further suggesting that the results generated by GEB are reliable and the logic/principles implemented in GEB are scientifically sound.

**Table 2:** The co-regulation T/Cs with expression profiles correlated to the T/C of "16 hr Pseudomonas infection" (A)^1^

T/C Name	Classification	Gene Number **(B)**^2^	Overlapping Gene Number **(C)**^3^	Overlapping Percentage **% (OP)**^4^	Correlation Coefficient **(CC)**^5^	Relation Index **(RI)**^6^
24 hr *Pseudomonas *infection	Pathogen infection	477	277	64	0.982917	0.623816

Leaf, WT, *Golovinomyces orontii *infection for 5 d vs. 0 d control	Pathogen infection	716	241	43	0.93683	0.385622

BTH Effect for 8 hr in *wrky18 *mutant	Plant defense elicitor	1245	296	36	0.943639	0.3242

BTH Effect for 8 hr in WT	Plant defense elicitor	1614	308	30	0.951557	0.279581

SA effect at 6 hr (Col-0)	Plant defense elicitor	421	121	30	0.954483	0.274902

*Pseudomonas syringae *pv *tomato *DC3000 hrcC-infiltration for 24 hr	Pathogen infection	1065	223	30	0.939357	0.272162

BTH treatment in *mil4 *mutant vs. H2O in *mil4 *mutant	Plant defense elicitor	652	184	35	0.872943	0.271469

BTH Effect for 24 hr in wrky18 mutant	Plant defense elicitor	1655	307	30	0.935343	0.263835

Leaf, *eds16*, *Golovinomyces orontii *infection for 5 d vs. WT, infection for 5 d	Plant defense related mutant	495	169	38	-0.82538	0.26286

*siz1-3 *mutant vs. Col-0 WT	Plant defense related mutant	987	221	32	0.889254	0.255498

*mil4 *overexpression line with BTH treatment vs. *mil4 *overexpression line control	Plant defense elicitor	574	181	37	0.820014	0.254887

SA effects at 4 hr (MT-0)	Plant defense elicitor	702	158	29	0.934091	0.254588

BTH Effect for 24 hr in WT	Plant defense elicitor	2062	341	27	0.94732	0.250527

*pmr5 pmr6 *double mutant vs. WT	Plant defense related mutant	423	126	31	0.892795	0.249832

Leaf, WT, *Golovinomyces orontii *infection for 7 d vs. 0 d control	Pathogen infection	2111	329	26	0.940966	0.23379

BTH treatment in WT vs. WT control	Plant defense elicitor	496	141	32	0.847156	0.230768

120 hr *Erysiphe orontii *infection	Pathogen infection	591	159	32	0.837777	0.229624

Whole plant, *mkk2*, 24 h BTH vs. *mkk2 *control	Plant defense elicitor	973	225	33	0.828929	0.228364

SA effect at 4 hr (Est)	Plant defense elicitor	259	78	24	0.96622	0.22756

PsmES4326 infection for 9 hr	Pathogen infection	340	99	27	0.90638	0.225606

Phytophthora infection for 24 hr	Pathogen infection	776	152	26	0.919965	0.222373

*Pseudomonas syringae *pv *phaseolicola *infiltration for 24 hr	Pathogen infection	1667	256	25	0.93104	0.216709

*upf3 *mutant vs WT	Others	635	123	24	0.938378	0.213205

Whole plant, WT, 24 h BTH vs. WT control	Plant defense elicitor	707	165	30	0.834236	0.211088

*Pst DC3118 COR-hrpS *double mutant infection 10 hr	Pathogen infection	418	90	22	0.963317	0.209057

Ozone effects	Plant defense elicitor	1544	247	25	0.898834	0.207327

SA effect at 4 hr (Tsu-1)	Plant defense elicitor	294	83	24	0.911413	0.204284

*cpr5scv1 *double mutant	Plant defense related mutant	742	163	29	0.833698	0.20177

Leaf, *eds16 *mutant, *Golovinomyces orontii *infection for 7 d vs. *eds16 *mutant, 0 d control	Pathogen infection	2644	341	22	0.939944	0.199188

Phytophthora infection for 12 hr	Pathogen infection	877	152	24	0.907767	0.199132

shoot under Caesium treatment	Others	187	64	22	0.938556	0.19851

E. coli *TUV86-2 fliC *mutant infection 7 hr	Pathogen infection	859	136	21	0.941937	0.194622

Whole plant, *mkk1*, 24 h BTH vs. *mkk1 *control	Plant defense elicitor	1003	176	25	0.867235	0.191285

SA effects at 4 hr (Van-0)	Plant defense elicitor	243	66	21	0.938173	0.18619

Pst DC3000 *hrpA *mutant infection 7 hr	Pathogen infection	796	121	20	0.947091	0.184426

*siz1-3 *mutant drought with treatment vs. Col-0 WT with drought treatment	Plant defense related mutant	1713	244	23	0.884965	0.182514

*Pseudomonas syringae *pv *phaseolicola *infiltration for 6 hr	Pathogen infection	1090	161	21	0.899435	0.177085

*upf1 *mutant vs WT	Others	268	85	26	0.82047	0.176332

WT (Col-0) Bgh infection vs. WT control	Pathogen infection	2489	296	20	0.924127	0.176158

6 hr control vs 0 hr control	Others	1128	154	20	0.924756	0.174548

shoot under potassium starvation	Others	1293	168	20	0.929743	0.173504

Pst DC3118 Coronatine infection 24 hr	Pathogen infection	483	82	18	0.955476	0.173289

*ataf1-1 *mutant, Bgh infection vs. *ataf1-1 *mutant control	Pathogen infection	3165	346	19	0.938368	0.171836

Phytophthora infection for 6 hr	Pathogen infection	1920	237	20	0.912724	0.171609

*S15-118 *mutant vs. WT	Others	160	63	23	0.854106	0.169901

Leaf, *eds16 *mutant, *Golovinomyces orontii *infection for 5 d vs. *eds16 *mutant, 0 d control	Pathogen infection	152	54	20	0.905124	0.166002

*35S::ERF104*, Flg22 treatment vs. *35S::ERF104*, control	Plant defense elicitor	1388	160	18	0.95289	0.164251

*pmr5 *mutant vs. WT	Plant defense related mutant	93	43	18	0.947634	0.16293

E. coli 0157:H7 infection 7 hr	Pathogen infection	582	88	18	0.940332	0.161603

SA effects at 4 hr (Kin-0)	Plant defense elicitor	237	59	19	0.919729	0.161515

*S58-2 *mutant vs. WT	Others	175	56	20	0.892642	0.160508

Leaf, *eds16*, *Golovinomyces orontii *infection for 7 d vs. WT, infection for 7 d	Plant defense related mutant	455	108	25	-0.78266	0.158267

Rosette leaf, flu mutant vs. WT	Others	1024	138	19	0.89576	0.157622

Triazolopyrimidine herbicide treatment vs. control	Herbicide	1768	191	17	0.938601	0.156599

Col-0 WT, Pst DC3000 (avrRpt2) infection vs. uninoculated control	Pathogen infection	629	110	21	0.827156	0.149031

*cpr5npr1svi1 *triple mutant	Plant defense related mutant	388	89	23	0.799919	0.14811

DC3000hrpA vs WT at 14 hr pathogen treatment	Pathogen infection	891	107	16	0.93812	0.148062

2 hr control vs 0 hr control	Others	864	113	18	0.898938	0.146689

OGs effects for 1 hr	Plant defense elicitor	866	122	19	0.86349	0.145894

AgNO3	Others	807	117	19	0.854076	0.143679

Rosette leaf, flu mutant, over-expressing *tAPX *vs. WT, over-expressing *tAPX*	Others	1414	149	16	0.926977	0.142656

Elicitor experiment, HrpZ treatment for 2 hr vs. 2 hr control	Plant defense elicitor	2043	211	17	0.905002	0.142587

Imidazolinone herbicide treatment vs. control	Herbicide	1843	176	15	0.948063	0.14226

flg22 effects for 1 hr	Plant defense elicitor	1714	180	17	0.908522	0.141837

*Pseudomonas syringae *pv *phaseolicola *infiltration for 2 hr	Pathogen infection	319	65	18	0.869464	0.140394

Pst DC3000 infection (5 hr) in wrky17 mutant	Pathogen infection	2918	271	16	0.918935	0.138735

Elicitor experiment, GST-NPP1 treatment for 4 hr vs. 4 hr control	Others	2044	214	17	0.882373	0.137416

high nitrogen effects	Others	1155	131	17	-0.89249	0.135869

Pst DC3000 infection (5 hr) in WT	Pathogen infection	2782	264	16	0.901735	0.135735

Whole plant, *mkk1/mkk2 *vs. WT	Plant defense related mutant	2559	241	16	0.905859	0.134531

Whole plant, mkk2, 24 h BTH treatment vs. WT, 24 h BTH treatment	Plant defense elicitor	262	55	17	0.886747	0.134518

Whole plant, mkk1/mkk2, 24 h BTH vs. WT 24 h BTH	Plant defense elicitor	1887	197	17	0.879539	0.134389

*gh3.5-1D *mutant Pst DC3000(avrRpt2) vs. *gh3.5-1D *un-inoculated control	Pathogen infection	2533	248	17	0.888302	0.134312

Elicitor experiment, Flg-22 treatment for 4 hr vs. 4 hr control	Plant defense elicitor	1259	134	16	0.906282	0.13422

senescence effects in pod	Senescence	1722	195	18	0.850641	0.134189

*sni1 *mutant	Plant defense related mutant	170	72	26	0.713915	0.1332

Pst DC3000 infection (5 hr) in wrky11 mutant	Pathogen infection	3067	276	16	0.909859	0.132532

Primisulfuron herbicide treatment vs. control	Herbicide	2805	242	15	0.931266	0.131749

^1^T/C of "16 hr Pseudomonas infection" has 1316 genes (A) that are significantly changed (2-fold and p-value 0.05 as cutoff)

^2^The number of the genes (B) that are significantly changed (2-fold and P value 0.05 as cutoff)

^3^The number of overlapping genes (C) between A and B

^4^The Overlap Percentage OP = 2*C/(A + B)

^5^The Pearson Correlation Coefficient (CC) of LOG2R of the overlapping genes

^6^The Relation Index RI = OP *C

Gene network building has been a hot research topic during the past few years [10,12,34,35]. GEB is not only able to construct gene networks based on the co-responsive relationship described above (Figure 6) but is also able to construct T/C networks based on the co-regulation relationship (Table 2). Another paper will address the details about constructing gene networks and T/C networks in *Arabidopsis*.

### Slide View

**Figure 7:**
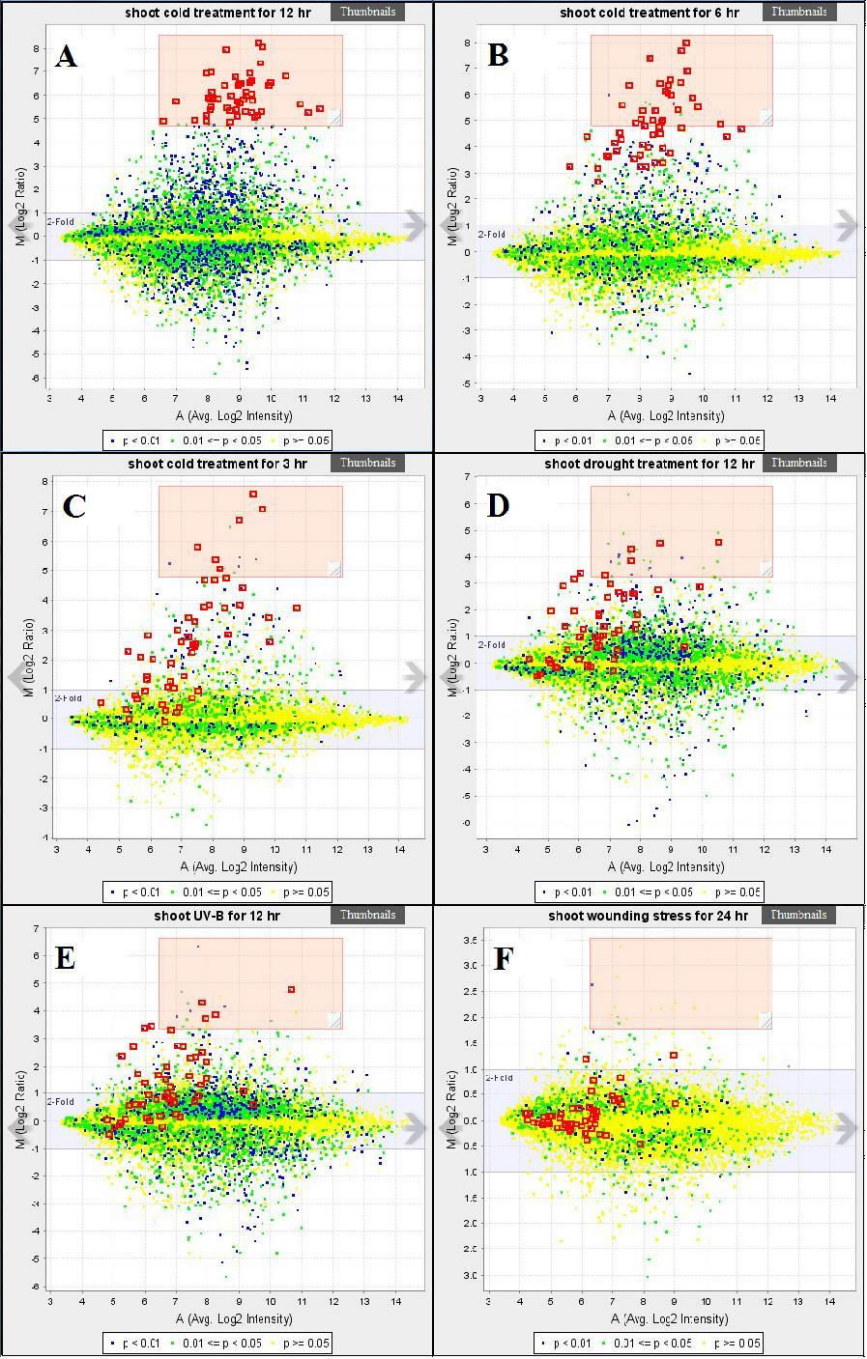
**A sample of Slide View for six T/Cs**. (3 cold treatments, 1 drought treatment, 1 UV-B treatment, and 1 wounding treatment). Users may test this function at http://www.expressionbrowser.com/arab/displayPairSlides.jsp?id=2055336&id=2055335&id=2055334&id=2055599&id=2055979&id=2056077.

The slide view of genes or T/Cs is designed to help users discover changes in multiple genes under various T/Cs or vice versa. Users can make a slide show to compare a set of T/Cs with multiple selected genes. For example, the user can search T/C conditions in Gene View (Figure 4) by typing "cold" in the search box and then selecting three T/C conditions with cold treatment of 12 hr, 6 hr, and 3 hr from the upper right box to the lower right box. After selecting the three "cold" conditions, the user can also search another three non-related T/Cs, such as "drought," "UV-B" and "wounding" with the same procedure. After the six necessary T/C conditions are selected, the user can click the "[slide]" link and then the six MA plots of T/Cs are shown as slides (Figure 7). In Figure 7A, the user highlights a certain number of genes by dragging, dropping and resizing the "highlighting window." A total of 51 genes with at least 30-fold increase (LOG2R > 4.9) in 12-hr cold condition are selected. To see how the selected genes are changed in other T/Cs, click the "next slide" arrow, and the next slide will appear. The selected genes in the first slide are still highlighted but the positions of the selected genes are changed in different slides. With this slide show, users are able to see the change of these 51 selected genes in different T/Cs. Figures 7B to 7F reveal changes in the selected genes in the T/Cs with treatments of 6-hr cold, 3-hr cold, 12-hr drought, 12-hr UV-B, and 12-hr wounding, respectively. These slides clearly demonstrate that the selected genes had highest fold changes under 12-hour cold treatment (Figure 7A). The fold-changes decreased in 6-hr (Figure 7B) and 3-hr (Figure 7C) cold treatments. The positions of the 51 selected genes in treatments of drought (Figure 7D), UV-B (Figure 7E) and wounding (Figure 7F) showed less similarity to "12 hr cold treatment". The Slide View is a very simple and powerful visualization tool for scientists to compare their candidate genes and see how the genes behave differently in the T/Cs across studies.

### Experiment View

Experiment View shows experiment title, description/design, lab information, samples, sample sets, biological replicates, and the definitions of T/Cs. This view helps users understand the data in detail. For example, the contents of the Experiment View of "Pathogen Series: Pseudomonas half leaf injection" can be seen at the following link http://expressionbrowser.com/arab/displayExperiment.jsp?id=2020113. There are three tabs in the Experiment View. The first tab, called "Details," displays experiment title, description, and other detailed information of the experiment. The second tab "T/C" contains information about the T/Cs in the experiment. The third tab "Samples and Data" contains information about all sample sets, samples and raw data files. Users can download raw microarray data files through the "Samples and Data" tab and then input these raw data into other microarray data-analysis software to analyze the data and to validate the results obtained from GEB.

## Conclusions

GEB is composed of a data extraction tool, a microarray data-management system, a data annotation tool, a data-processing pipeline, and a search & visualization tool. The heterogeneity of diverse experimental designs has been greatly mitigated by re-organizing different experimental treatment levels into T/Cs so that cross-experimental data integration is easily achieved. GEB separates data processing from interactive display. It pre-processes data and generates data plot images, and then displays the processed data with a web2.0-based interactive user-interface, according to users' requests. This design allows heavy computing to be done offline, and thus allows a large number of data points to be queried quickly and displayed interactively in real-time. GEB displays all data points in one view so that users do not need to scroll down display pages to obtain the trend or pattern of gene expressions from all data points. The highlighting and searching functions in Gene View, T/C View, and Slide View greatly facilitate dynamically exploring the data points based on users' interests. As an additional strategy to improve usability, all raw data and calculated data in GEB are accessible via a full-text search engine. GEB also computes relations of co-regulation T/Cs and co-responsive genes. These relations are the foundation for building gene networks and T/C networks.

## Availability and requirements

• Project Name: Gene Expression Browser (GEB)

• Public web service: http://www.ExpressionBrowser.com Free and no registration.

• Programming Language: Java, R

• Database: MySQL

• Software License: The software license is owned by GeneExp. GeneExp grants free licenses to non-profit organizations and general licenses to commercial organizations.

• License request: support@ExpressionBrowser.com

## Abbreviations

A: Average Log2 Intensity; AJAX: Asynchronous JavaScript and XML; ATH1: A light-regulated Arabidopsis thaliana homeobox 1 gene; BTH: Benzothiadiazole; CC: Correlation Coefficient; CIBEX: Center for Information Biology Gene Expression Database; CPU: Central Processing Unit; EDS: Enhanced Disease Susceptibility; EBI: The European Bioinformatics Institute; GDS: Granite Data Services; GEB: Gene Express Browser; GEO: Gene Expression Omnibus; JPEG: Joint Photographic Experts Group; LOG2R: Log2 Ratio of Treatment over Control; MA plot: a Quick Overview of Intensity-dependent Ratio of Microarray Data; M: LOG2R; N: Nitrogen; NASC: Nottingham *Arabidopsis *Stock Center; NCBI: The National Center for Biotechnology Information; NIG: The National Institute of Genetics; *PR *gene: Pathogenesis-related gene; R gene: Resistance genes; RI: Relation Index; RMA: Robust Multichip Average; SA: Salicylic Acid; SAR: Systemic Acquired Resistance; TAIR: Texas Association for Institutional Research; T/C: Treatment over Control; %CV: Percentage Coefficient of Variation; OP: Overlapping Percentage; UV-B: Ultraviolet-B Radiation.

## Authors' contributions

MZ, XF, ST proposed software requirements. MZ, YZ, XF, MSK did software specification and design. YZ developed statistical protocols. MZ designed database schema and developed computational algorithms and the software. LL, LY, ST, JT, WY, YA tested the software application and wrote the manual. MZ downloaded and processed raw microarray data from GEO and NASC. LL, LY, ST, JT, WY annotated the data and nominated the T/Cs. MZ, YZ, XF, ST, MSK, YA drafted the manuscript. MZ, YZ, XF, LL, LY, ST, JT, WY, MSK wrote different parts of the manuscript. MZ, YZ, XF, MSK, YA assembled all parts written by different authors together into this manuscript. All authors read and approved this manuscript.
